# A self-sustaining serpentinization mega-engine feeds the fougerite nanoengines implicated in the emergence of guided metabolism

**DOI:** 10.3389/fmicb.2023.1145915

**Published:** 2023-05-15

**Authors:** Michael J. Russell

**Affiliations:** Dipartimento di Chimica, Università degli Studi di Torino, Torino, Italy

**Keywords:** Acetothermia, double layer oxyhydroxides, entropy disproportionation, fougerite/green rust, serpentinization, submarine alkaline vent theory (AVT)

## Abstract

The demonstration by Ivan Barnes et al. that the serpentinization of fresh Alpine-type ultramafic rocks results in the exhalation of hot alkaline fluids is foundational to the submarine alkaline vent theory (AVT) for life’s emergence to its ‘improbable’ thermodynamic state. In AVT, such alkaline fluids ≤ 150°C, bearing H_2_ > CH_4_ > HS^−^—generated and driven convectively by a serpentinizing exothermic mega-engine operating in the ultramafic crust—exhale into the iron-rich, CO_2_> > > NO_3_^−^-bearing Hadean ocean to result in hydrothermal precipitate mounds comprising macromolecular ferroferric-carbonate oxyhydroxide and minor sulfide. As the nanocrystalline minerals fougerite/green rust and mackinawite (FeS), they compose the spontaneously precipitated inorganic membranes that keep the highly contrasting solutions apart, thereby maintaining redox and pH disequilibria. They do so in the form of fine chimneys and chemical gardens. The same disequilibria drive the reduction of CO_2_ to HCOO^−^ or CO, and the oxidation of CH_4_ to a methyl group—the two products reacting to form acetate in a sequence antedating the ‘energy-producing’ acetyl coenzyme-A pathway. Fougerite is a 2D-layered mineral in which the hydrous interlayers themselves harbor 2D solutions, in effect constricted to ~ 1D by preferentially directed electron hopping/tunneling, and proton Gröthuss ‘bucket-brigading’ when subject to charge. As a redox-driven nanoengine or peristaltic pump, fougerite forces the ordered reduction of nitrate to ammonium, the amination of pyruvate and oxalate to alanine and glycine, and their condensation to short peptides. In turn, these peptides have the flexibility to sequester the founding inorganic iron oxyhydroxide, sulfide, and pyrophosphate clusters, to produce metal- and phosphate-dosed organic films and cells. As the feed to the hydrothermal mound fails, the only equivalent sustenance on offer to the first autotrophs is the still mildly serpentinizing upper crust beneath. While the conditions here are very much less bountiful, they do offer the similar feed and disequilibria the survivors are accustomed to. Sometime during this transition, a replicating non-ribosomal guidance system is discovered to provide the rules to take on the incrementally changing surroundings. The details of how these replicating apparatuses emerged are the hard problem, but by doing so the progenote archaea and bacteria could begin to colonize what would become the deep biosphere. Indeed, that the anaerobic nitrate-respiring methanotrophic archaea and the deep-branching *Acetothermia* presently comprise a portion of that microbiome occupying serpentinizing rocks offers circumstantial support for this notion. However, the inescapable, if jarring conclusion is drawn that, absent fougerite/green rust, there would be no structured channelway to life.

## 1. The submarine alkaline vent theory

The three founding facts underpinning the submarine “alkaline vent theory” for the emergence of life are:

1. Barnes and O’Neil’s (1969) conclusion that: “convecting seawater at < 200°C would have serpentinized the crust, becoming alkaline by this process of hydrolysis, much as today, hot springs involving ground-water circulating in ophiolite, have a pH of between 11.5 and 12″;
2. Dudley Foster’s iconic photograph of the acidic ~ 380°C Black Smoker hydrothermal chimney with polychaete and tube worms on the East Pacific Rise (Ballard and Grassle, 1979; Spiess et al., 1980);
3. The smaller scale hydrothermal chimneys, chemical garden spires, and microbialites discovered in the ~ 340 million year old ore deposits in Ireland, inspired by the “Black Smoker” reports (Larter et al., 1981; Russell, 1996).

Jack Corliss et al. had calculated, on the basis of geochemical studies of basalts from the Mid-Atlantic Ridge and from the silica and magnesium chemistry of warm springs exhaling from the Galapagos submarine ridge, that 300°C metal-bearing hot springs should be found at ocean floor spreading centers (Corliss, 1971; Corliss et al., 1979). The discovery of acidic Black Smokers teaming with life met these predictions and led Corliss, John Baross and Sarah Hoffman to formulate a “hydrothermal origin-of-life hypothesis.” Rejected from *Nature* and *Science*, they resorted to the “grey literature” to present their manuscript (Corliss et al., 1980, 1981; Baross and Hoffman, 1985; Levitt, 2023). As reported in Corliss et al. (1980), the hypothesis maintains that “Submarine hydrothermal systems provide all of the conditions necessary for the abiotic synthesis of organic compounds, polymers, and simple cell-like organisms. The continuous flow of circulating fluids in a hydrothermal system provides the thermal and chemical gradients which create the variation in conditions necessary for the successive reactions to take place. Other models for the origin of life fail to fulfill one or more of these requirements.”

This “anaerobic chemoautotrophic” hypothesis riled those who had accultured to the Oparin–Haldane–Urey–Miller dogma of how life originated (Lahav, 1985; *cf.*, Lane et al., 2010). Indeed, Stanley Miller himself, with his colleague Jeffrey Bada, took to print in 1988, opining “The high temperatures in the vents would not allow synthesis of organic compounds, but would decompose them, unless the exposure time at vent temperatures was short… Even if the essential organic molecules were available in the hot hydrothermal waters, the subsequent steps of polymerization and the conversion of these polymers into the first organisms would not occur as the vent waters were quenched to the colder temperatures of the primitive oceans” (Miller and Bada, 1988). Their criticism prompted a response, in which it was argued from the discovery of fossil chimneys at Silvermines and the Tynagh base-metal ore deposit in Ireland (Larter et al., 1981; Boyce et al., 1983; Banks, 1985) that “similar, less extreme environments are known and could have provided suitable sources of chemical energy and nutrients as well as stable ‘culture chambers’” (Russell et al., 1988).

**Figure 1:**
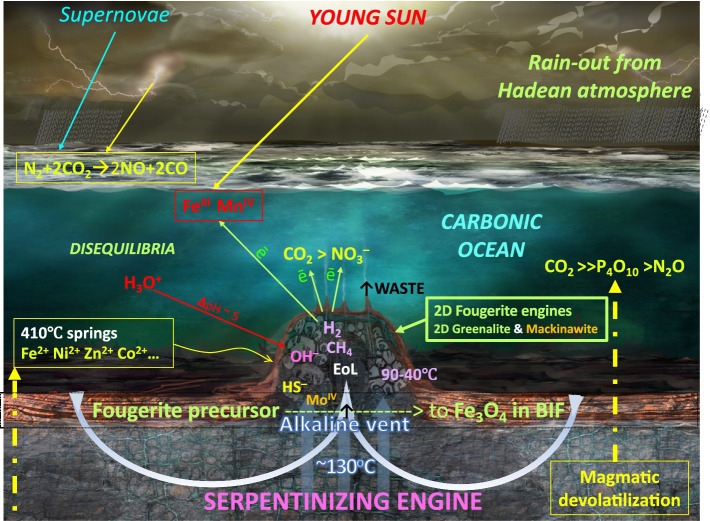
Initial conditions responsible for the emergence of life (EoL) according to the alkaline vent theory (AVT; Russell et al., 2013). Mantle-derived volcanic emanations (> 700°C) and high temperature acidic hot springs (~410°C) inject CO_2_, P_4_O_10_, some N_2_O, and the transition elements directly through the Hadean ocean floor into the cool carbonic Hadean ocean (Yamagata et al., 1991; Javoy and Pineau, 1991; Macleod et al., 1994; Kasting and Siefert 2001; Wong et al., 2017; Mandon et al., 2019; Ueda and Shibuya, 2021; Brady et al., 2022; Buessecker et al., 2022; Heays et al., 2022; Nishizawa, 2022; Tatzel et al., 2022). At the ocean bottom, and diffusing laterally and upward, these volatiles and ions remain as saturated or supersaturated until meeting with alkaline hydrothermal solutions (at ≤ 130°C) produced by the serpentinization engine (Barnes and O’Neil, 1969; Russell et al. 1989; Branscomb and Russell, 2018; Shibuya and Takai., 2022). Fougerite, along with subordinate amorphous silica, greenalite, and subsidiary mackinawite spontaneously precipitate at the interface between the alkaline solution and the ocean solvent (Russell, 2018; Tosca et al., 2016, 2019; Borrego-Sánchez et al., 2022). These inorganic barriers maintain the pH and redox disequilibria that drive the emergence of life (EoL; Russell and Hall, 1997), so focusing the electrochemical disequilibria as native electrons, cations, anions, and dissolved gasses across the fougerite exteriors of the mound. Thus, these nanocrysts, assisted by the electron-conducting mackinawite, are forced into acting as nanoengines to resolve the disequilibria and thereby bring embryonic life into being (Arrhenius, 2003; Mielke et al., 2010; Nitschke and Russell, 2012, 2013; Barge et al., 2015a; Halevy et al. 2017; Russell and Nitschke, 2017; Wong et al., 2017; Yamamoto et al., 2017, 2022; Kitadai et al., 2018; Duval et al. 2019; Ooka et al., 2019; Hudson et al., 2020; Nitschke et al., 2022; Buessecker et al. 2022). [NB., The electron acceptors and the H^+^ shown to be dissolved in the ocean are constantly delivered by the venturi effect to the outer barrier of the mound (Russell and Hall, 1997)]. Redrawn from Branscomb et al. (2017). An extensive aureole of the same minerals surround this and other vents, now altered to banded iron formation (BIF; Konhauser et al., 2007; Pons et al., 2011; Mloszewska et al., 2012). Not to scale. The serpentinite photograph was generously provided by Laura Barge.

However, a reading of Ivan Barne’s studies and our field studies on Alpine-type ultramafic rocks in Southern Europe and Turkey (e.g., Fallick et al., 1991) led us to propose a substitution of the acidic hydrothermal spring models responsible for Black Smokers and exhalative orebodies, with a serpentinization-driven alkaline hydrothermal model that more appropriately explained the sources of fuel to feed life’s ‘origin’ (Russell et al., 1989). Further development of the model had the alkaline hydrothermal solutions precipitating iron sulfide bubbles on contact with “the mildly oxidized, acidic and iron-bearing Hadean ocean water” (Russell et al., 1993). While this model could demonstrate the analogy with CO_2_-based autotrophic metabolism—more fundamental was its explanation of the otherwise enigmatic origin of Peter Mitchell’s *proton motive force* (PMF). How life could have invented the PMF had been a puzzle, so a key insight of AVT was that no invention of the ‘force’ was necessary—the PMF had been there from the beginning, freely developed from one aspect of the initial conditions, i.e., as a proton gradient imposed across mineral precipitate membranes—itself a result of the acidulous ocean interfacing the alkaline hydrothermal fluid as it exhales from the Hadean ocean floor (Mitchell, 1961; Russell et al., 1994; Figure 1).

Competing with AVT, though assuming only acidic conditions, is Günter Wächtershäuser’s theory that the origin of life involves the reduction of copious volcanic CO_2_ through the reductive acetyl coenzyme-A pathway to acetate (Wächtershäuser, 1988)—the pathway that is now broadly, but not universally, accepted for this role (Huber and Wächtershäuser, 1997; Peretó et al., 1999; Martin and Russell, 2003, 2007; Say and Fuchs, 2010; Martin, 2020; Boyd et al., 2023). That life was first engaged in the reduction of oceanic CO_2_ through the acetyl coenzyme-A pathway was incorporated in AVT and expressed in the (grossly) oversimplified empirical Equation 1 (Russell and Martin, 2004)


(1) $$2{\text{CO}}_{2}+\text{HSCoA}+4{\text{H}}_{2}\to {\text{CH}}_{3}\text{COSCoA}+3{\text{H}}_{2}\text{O}$$

But more recently, we have argued for an alternative whereby the disequilibria across a ferrous–ferric oxyhydroxide membrane—with FeS now as a subordinate—are several and the potentials significantly greater, namely, the ‘denitrifying methanotrophic acetogenic pathway’ (DnMAP; Ducluzeau et al., 2009; Nitschke and Russell, 2013). This suggestion arises: (1) in consideration of methane emanations accompanying H_2_ in some serpentinizing systems—an overlooked otherwise ‘wasted’ fuel and source of organic carbon (Kelley, 1996); (2) the ‘energetic’ requirement for an electron acceptor with a higher potential than CO_2_ to order protometabolism (Russell and Hall, 1997; Nitschke and Russell, 2013); (3) in choosing the methanotrophic route, the steep uphill ‘free’ energy climb to the highly unstable formyl intermediate in the classic acetyl coenzyme-A pathway is avoided (Maden, 2000; Stojanowic and Hedderich, 2004); (4) to provide a source of ammonium, which otherwise is lacking (Nitschke and Russell, 2013); (5) and the ungainliness of the classic pathway’s two ‘legs’, i.e., the disproportionate numbers of the reductive steps involved, one to reduce CO_2_ to CO or formate, as against six to reduce it to a reactive methyl sulfide entity, is replaced by the more symmetrical, less complicated ‘CO_2_-reducing and denitrifying methanotrophic pathways’ that converge to acetyl coenzyme-A (DNitAP; Nitschke and Russell, 2013). This pathway is also highly simplified to


(2) $$\begin{array}{l} {\left\{{4H}^{+}+{\text{CO}}_{2}+N_{2}O\right\}}_{\text{ocean}}+{\left\{8H\cdot \;+\;{\text{CH}}_{4}+{\text{HS}}^{-}+{\text{OH}}^{-}\right\}}_{\text{hydrothermal}}\\ \to {\left\{{\text{CH}}_{3}\text{COSH}+{2\text{NH}}_{4}{\;}^{+}\right\}}_{\text{metabolism}}+3_{H2}O_{\text{waste}} \end{array}$$

Apart from the addition of methane, this alternative takes into account the disequilibria focused at a submarine alkaline vent as outlined in Russell and Hall (1997): the natural proton motive force and a ‘respiratory’ redox mechanism with electrons (some of them bifurcated) that H_2_, *via* 2H•, provides, processing through a green rust (fougerite) nanoengine as electrons are conducted to high potential electron acceptors, e.g., nitrate (Nitschke and Russell, 2012, 2013; Barge et al., 2015a; Russell and Nitschke, 2017; Wong et al., 2017; Buessecker et al. 2022; Nitschke et al., 2022). We reiterate that this reductive mechanism also accounts for the required on-site source of ammonium for amino and nucleic acid synthesis, otherwise far from obvious (Barge et al., 2019).

Yet, a further change to AVT was the adoption of the term ‘emergence’, as we came to understand that the conventional ‘origin of life’ was an idiom devoid of evolutionary connotation (Russell et al., 1993)—this from the readings of Prigogine and Stengers’ (1984) book “Order out of Chaos” and Wicken’s (1987) volume “Evolution, Thermodynamics and Information.” Indeed, contemplating life’s ‘emergence’ forced a more serious consideration of the role of serpentinization in life’s onset (Russell et al., 2013).

In the present theoretical contribution, life’s emergence is traced from serpentinization to its fledging as a dynamic system that dramatically reduces entropy (thus substantially increasing the rate at which the driving disequilibria produce entropy). We conclude that the first proto-metabolic steps take place in the natural chemical garden spires that developed at a submarine alkaline hydrothermal vent sometime in the ~ 500 million year span of the Hadean era. Exothermic serpentinization is the mega-engine operating within the ultramafic oceanic crust that works to drive alkaline hydrothermal convective systems bearing H_2_ > CH_4_ fuels to exhale into the then carbonic, phosphate, nitrate, NO, and N_2_O as well as metal complex-, and proton-bearing acidulous ocean—the disequilibria resulting in autotrophic metabolisms involving quinone-dependent NO reductase and membrane-bound N_2_O reductase—supporting a primitive aerobic respiration (Ducluzeau et al., 2009, 2014; Nitschke and Russell, 2013; Russell et al., 2013; Brady et al., 2022; Buessecker et al., 2022; Farr et al., 2022).

This is where the structural and ionic complexity of the 2D green rust mineral fougerite (~Fe^2+^_4_Fe^3+^_2_(OH)_12_CO_3_.3H_2_O) moves to center stage in AVT. Initially precipitated as white rust (amakinite) under alkaline conditions, the oxidation by water to fougerite—with the concomitant evolution of H_2_—is rapid (Trolard et al., 2022; Helmbrecht et al., 2022). Though more ordered, i.e., of lower entropy than those of the contributing solutes, amakinite/fougerite precipitation is entropy-driven (Van Santen, 1984). The structure of fougerite (formerly green rust) is complex but not “pre-designed,” i.e., its growth is not algorithmic, it is merely self-ordered and requires no prescription (*cf.* Arrhenius, 2003; Abel and Trevors, 2006). It is the only macromolecular entity known to us with the chemical and physical flexibility and potential to respond to disequilibria at the vent. Thus, we propose that it has the wherewithal to act as the nanoengine to impel life into being. The iron sulfides mackinawite (FeS) and greigite (Fe_3_S_4_)—as subordinate components of the hydrothermal chimneys and spires—still hold vital support roles in their ‘free energy’ converting capacities and as conductors and semiconductors in AVT (Nitschke and Russell, 2009; Vasiliadou et al., 2019; Hudson et al., 2020).

## 2. Serpentinization—life’s mother engine

The casting of exothermic serpentinization of ocean crust as a disequilibria- (“free energy”-) converting mega-engine (Russell et al., 2013) is based on an extensive literature (Barnes and O’Neil, 1969; Barnes et al., 1978; Neal and Stanger, 1983, 1984; Fallick et al., 1991; Kelley et al., 2001; Lowell and Rona, 2002; Russell and Arndt, 2005; Mielke et al., 2010; Paukert et al., 2012; Russell et al., 2013; Branscomb and Russell, 2018). Inspiration was rooted in the physics of materials, as considered by Cottrell (1979), whereby mechanical stress is converted through feedbacks, as in an engine, into physical and chemical disequilibria such as to result in “living things,” themselves engines. How might we see such engines developing in the early Earth?

With Hadean days so short, the moon so close, and the Earth’s mantle so soft, the mafic to ultramafic oceanic crust suffers pulses of incessant cracking, jointing, faulting, and brecciation that allow the invasion and gravitation of cool ocean water to depth (Miller et al., 2016; Heller et al., 2021; Tatzel et al., 2022). Once cracks form in a tensile stress regime near the surface, feedback ensures that smaller stresses are required to keep them ratcheting down through the crust as exothermic cracking engines (Cottrell, 1979; Lowell and Rona, 2002). Furthermore, the hydrostatic pressure so imposed increases the effective stress, though only after the crack has propagated at the nanoscale, feeding back to further cracking while the elasticity at the tip is converted to ‘free energy’—a counter-intuitive realization (Cottrell, 1979). Such an autocatalytic feedback is further augmented by the hydrostatic pressure imposed on the mafic to ultramafic wallrock by the ocean waters gravitating to depth—a pressure that increases both the effective tensile and related sheer stresses (Cathles, 1990; Russell and Skauli, 1991). These couplings eventuate in the cracks reaching brittle-to-ductile transition zones in ultramafic rocks estimated from the hydrogen isotope work of Proskurowski et al. (2006) to bottom out at ~ 150°C, corresponding to an eventual maximum crustal depth of around 8–10 km (Macleod et al., 1994). This self-ordering and self-healing process continues until much of the upper crust is hydrated and carbonated, causing a lowering of density (≳ 2.6 g cm^−3^) compared to the antecedent unaltered ultramafic crust (3.3 g cm^−3^), through expansion allowed for by crustal extension and/or domal uplift in processes leading to further cracking (Fujioka et al., 2002; Pons et al., 2011).

The exhaust from the serpentinizing system—as heat and solutes—is discharged in convective hydrothermal alkaline updrafts buffered to a pH of 10–11 units guided by approximately vertical fractures in a process that lasts a minimum of 30,000 years (~ 10^21^ nanoseconds; Früh-Green et al., 2003). The tectonic, thermal, and chemical disequilibria are resolved through hydrothermal convection to result in a hydrothermal fluid—initially carbonic ocean water—by being reduced to H_2_ and short carboxylic acids and sparse methane (Früh-Green et al., 2003; Proskurowski et al., 2006; Ludwig et al., 2011; Tutolo et al. 2020; White et al., 2020; Albers et al., 2021; Figure 1; Equation. 3):


(3) $$\begin{array}{l} {\left\{12{\text{Ca}}_{0.25}{\text{Mg}}_{1.5}{\text{Fe}}_{0.25}{\text{Si}}_{2}O_{6}\right\}}_{\text{augite}}+16H_{2}O\to \;\;\\ {\left\{{6\text{Mg}}_{3}{\text{Si}}_{2}O_{5}{\left(\text{OH}\right)}_{4}+12{\text{SiO}}_{2}+{\text{Fe}}_{3}O_{4}\right\}}_{\text{serpentinite}}+\;\\ +{3\text{Ca}}^{2+}+{6\text{OH}}^{-}+H_{2}↑ \end{array}$$

Tidal and seismic pumping are additional inputs to the workings of this complex engine (Sibson et al., 1975; Davis and Becker, 1999; Glasby and Kasahara, 2001).

Spasmodic charges of methane, as well as of formate and acetate, are also recorded, both by a direct analysis and in laboratory experiments (Shock, 1992; Windman et al., 2007; White et al., 2020). However, it may be that much of the methane is derived through leaching from that generated in the lower crust and entrained in the same solutions (Shock, 1992; White et al., 2020). This thermal and chemical waste from serpentinization is now transported to the ocean floor in a hydrothermal solution that finds itself well out of thermal and electrochemical equilibrium with its new host, the iron-rich carbonic ocean water from which it first derives (Figure 1). Furthermore, the immediate effect of the meeting of the two contrasting solutions is the spontaneous precipitation of iron oxyhydroxides accompanied by silica and minor iron sulfides (Barge et al., 2015a,b, 2020; Helmbrecht et al., 2022). A portion of the precipitates makes up the hydrothermal chimneys and spires, while entrained flocs escape from this, and other alkaline springs, to disperse and lithify to banded iron formations comparable to those in Isua in the early Archean of western Greenland and in the Hadean Nuvvuagittuq greenstone belt in Canada (Appel, 1980; Papineau et al., 2011; Pons et al., 2011; Mloszewska et al., 2012; Halevy et al., 2017; Tosca et al., 2019; Bindeman and O’Neil, 2022).

**Figure 2:**
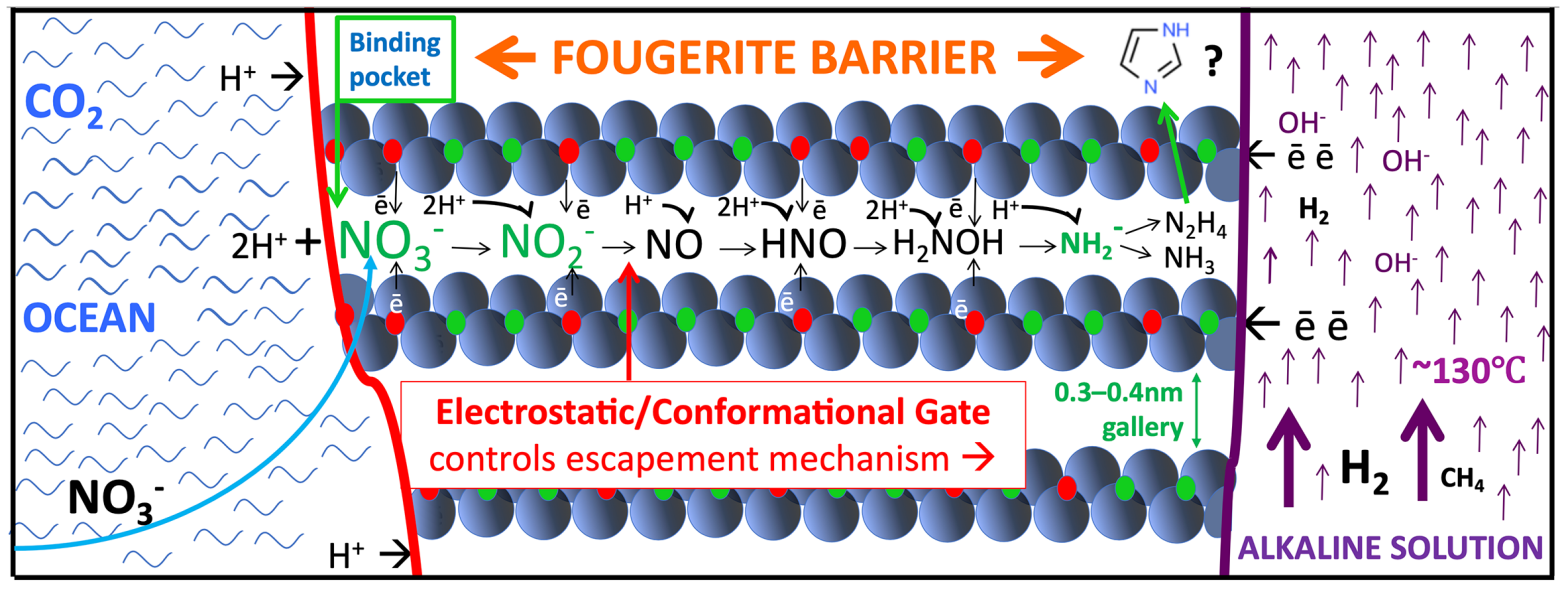
Fougerite modeled as a ready-made multifunctional motor enzyme/pump precursor set in the inorganic membrane, wherein it reduces nitrate drawn from the ocean (curved blue arrow to the left) to aminogen or ammonium, or nitrite to NO, N_2_O, and N_2_, vectored from ‘left’ to ‘right’ within the hydrate galleries (Hansen et al., 2001; Génin et al., 2005, 2006; Trolard et al., 2007, 2022; Trolard and Bourrié, 2012; Gerbois et al., 2014; Russell, 2018; Duval et al., 2019, 2020). At the same time and in theory, methane would be converted to a methyl group by NO (Kampschreur et al., 2011; Nitschke and Russell, 2013). Barge et al. (2019, 2020) show that in the same circumstances, pyruvate can be aminated to alanine and oxalate to glycine. Hydrazine is another speculative product (Duval et al., 2020). Note that an anion-binding pocket forms by the oxidation of the opposed iron molecules as they are confronted with nitrate which is thereby reduced (Nitschke and Russell, 2013). However, transmission (‘escape’) of a product to the interior is only permitted (ungated) when the nitrite is itself reduced to neutral NO and can be driven by the ionic gradients further into the interlayer. Note too that the flows (and counterflows) are vectorial, controlled by electron hopping rates (Wander et al., 2007). There is an expectation that short peptides will be produced within the interlayers (Muñoz-Santiburcio and Marx, 2017; Erastova et al., 2017; Grégoire et al., 2018; Holden et al. 2022) and partially extruded into the spire’s interior. Further H_2_ and organic molecules can be released to the hydrothermal flow by delamination and/or diagenetic alteration of fougerite to magnetite at depth in the mound (Asimakidou et al., 2020; Farr et al., 2022). The upward-directed arrows to the right signify the alkaline hydrothermal updraft, and the smaller arrows either side of the fougerite representing the inorganic membrane denote the direction of electron and proton flow. Not to scale.

The membranes precipitated at the hydrothermal mound have the effect of frustrating the release of the pent-up disequilibria, until a weakness can be found to guide interaction of the contrasting fluids. In the case of serpentinization and convection—the mega-engines just described—this was *via* chance cracks in the crust. However, at the nanoscale, the dissipative system finds a way to partial relaxation by the forced exploitation of nanochannels prized from hydrous cleavage cracks constituting fougerite interlayers (Figure 2). Furthermore, the redox- and pH-active nanochannels within the interlayers might impose a vectorial two-way ordering—a primitive guidance system—along the reductive and oxidative synthetic steps of the denitrifying methanotrophic acetogenic pathway toward an incomplete reverse tricarboxylic acid cycle (TCA) (Hartman, 1975; Wander et al., 2007; Nitschke and Russell, 2013; *cf.*, Gatenby and Frieden, 2017; Figure 2).

Thus, a way is open, even at the nanoscale for a specialized dissipative (entropy-generating metabolizing) engine to materialize in obeyance to the Universe’s predilection to ‘produce’ ever more disorder in its blind bid to continue its relaxation, independent of scale, from its initial excruciating disequilibrium at the origin of space–time (Nitschke and Russell, 2010; Russell et al., 2013; Carroll, 2016).

## 3. Life is, and was at its emergence, a disequilibria-converting macromolecular nanoengine

The series of orderly convection engines governed by physical transitions in the body of our planet, brought to a head by the serpentinization cracking engine, ultimately results in a long-lasting flow of reduced alkaline fluids into a highly contrasting relatively oxidized and mildly acidic Hadean seawater. Furthermore, the interactions of two solutions at the spontaneously precipitated membrane provide just the electrochemical disequilibria required to drive entropy-reducing metabolic pathways and reproductive cycles (Hitchcock and Lovelock, 1967; Russell and Hall, 1997; Russell and Arndt, 2005; Nitschke et al., 2022). However to bring these factors into play, disequilibria-converting engines are again required. Yet, the building components for construction are necessarily restricted to any inorganic materials at hand. Of course, at every step, engines must locally disproportionate entropy in such a ratio as to ensure its decrease in the driving of anabolic metabolism by an overall larger increase in entropy. This is achieved by the transportation of uncooperative molecules as waste from the partially open protometabolizing system.

Glaringly obvious is the requirement for the coupled hydrolysis of adenosine triphosphate, ATP, in present-day life—and thereby the need for its synthesis (Whicher et al., 2018; Pinna et al., 2022). To achieve such biosynthesis, Mitchell (1961) showed a pH gradient—a proton motive force—to be capable of driving the condensation of adenosine diphosphate (ADP) and inorganic phosphate (Pi) to adenine triphosphate (ATP) *via* the complex enzyme ATP synthetase situated in life’s membranes. Thus, Mitchell apparently dispensed with the long-favored view that a ‘high energy’ intermediate molecule was responsible, calling his process ‘chemiosmosis’. However, Boyer (1997) first made mechanical sense of Mitchell’s finding, demonstrating that ATP synthetase is actually a rotating nanoengine involving ‘binding-change’ and gated escapement mechanisms driven by the proton gradient. In life, protons are delivered by the machinations of complex 1 and the like (Hedderich, 2004; Kaila, 2021). Boyer mapped out the stages the ATP synthetase rotatory enzyme took to complete the cycle, realizations now fundamental to an understanding of how life works and indicating how some, albeit simpler engine must have worked from the very beginning (Boyer, 1979, 1997; Astumian, 2012; Astumian et al., 2016; Anashkin, et al., 2021). For reasons of pedagogy, Yoshida et al. (2001) compare the ATPase to a Wankel engine, while Carter (2020) likens the binding change and reciprocally coupled gating mechanism to escapements in the workings of a mechanical clock.

Important though ATP is, clearly ATP synthetase itself is much too complicated to have been available at life’s onset. Indeed, the discovery by Baltscheffsky et al. (1966) that inorganic pyrophosphate (PPi), situated in the membrane, can act as ‘energy donor’ in an electron-transport phosphorylation system introduced the hypothesis that a proton pyrophosphatase (H^+^-PPase) was a precursor to the ATP (Russell et al. 1994; Baltscheffsky et al., 1999). These reversible vacuolar pyrophosphatases are stochastic nanopumps (Lin et al., 2012, Li et al., 2016), in which a highly restricted number of water molecules within the axial region have the effect of dampening the flow of protons or cations. The protons make their way one-by-one through the gating mechanism, either inward or outward, depending on the ambient disequilibria (Branscomb and Russell, 2013, 2019; Scholz-Starke et al., 2019; Astumian et al., 2020).

However, PPi has strong competitors as a phosphorylating agent, e.g., acetyl phosphate [AcP]. Acetyl phosphate itself is readily generated from thioacetate and disodium phosphate under alkaline conditions (Heinen and Lauwers 1996; Huber and Wächtershäuser, 1997; Whicher et al., 2018). AcP is especially attractive as a precursor candidate of ATP (Pinna et al., 2022). Furthermore, Whicher et al. (2018) demonstrate the phosphorylation of ribose to ribose-5-phosphate and the phosphorylation of ADP to ATP also under alkaline conditions—the only nucleoside diphosphate to be thus phosphorylated—so explaining the primacy of ATP in bioenergetics (Pinna et al., 2022). Given that Wang et al. (2019) produce PPi from AcP and Pi, but not from 2Pi across an Fe-rich membrane in a microfluidic rig, would leave AcP as, perhaps, the major phosphate player in early metabolism. However, there are still other compounds such as the linear oligophosphates, glycolaldehyde phosphate (Arrhenius et al., 1993; Pitsch et al., 1995; Krishnamurthy et al., 1999), and the cyclic trimetaphosphate (TMP) shown by Etaix and Orgel (1978) to be capable of phosphorylating nucleosides in water—a discovery transferred into the 2D interlayers of the double-layer oxyhydroxides (DLHs; Yamanaka, 1988; Kuma et al., 1989; Yamagata et al., 1997; Kolb et al., 1997). Kolb et al. (1997), using TMP, induce phosphorylation of the glycolate ion in the interlayers of DLHs to glycolophosphate and diphosphate at a rate which is independent of the external concentration of glycolate ion in the range of 1–100 mM as measured—a remarkable and highly significant finding as we shall see. The phosphorylation can be followed by measuring the height of the interlayer; the initially absorbed hydroxyl height measures 0.29 nm, the glycolate replacement is 0.49 nm, and the trimetaphosphate (TMP) on its own is 0.68 nm, while adding TMP to glycolate to produce glycolophosphate plus the diphosphate generates a height of only 0.64 nm (Adam and Delbrück, 1968; Kolb et al., 1997).

Distinct from, and additional to the pyrophosphatase argument, and following Wächtershäuser (1990) and Peretó et al. (1999), the direct reduction of carbon dioxide *via* the reductive acetyl coenzyme-A pathway was next considered, though in terms of an inorganic FeS membrane rather than through the pyrite reaction (Russell and Martin, 2004). Branscomb and Russell (2013) modeled such a reduction involving the hydrogenation of CO_2_ dissolved in, and sourced from, the most ancient ocean, combined with that ambient proton force in a membrane comprising iron monosulfide and fougerite. Several authors calculate that the proton motive force summing to 2 pH units or more (equivalent to ~ 120 + mV) will facilitate the reduction of CO_2_ in alkaline waters (Russell and Hall, 1997; Schoepp-Cothenet et al., 2013; Sojo et al., 2016). Since then, and following Vasiliadou et al. (2019), Reuben Hudson and his coworkers have tested the latter hypothesis which is of direct relevance to the AVT using a microfluidic technique involving an iron sulfide membrane, duly demonstrating the requirement for “proticity” in such a reduction (Hudson et al., 2020). Whether fougerite, lightly dosed with sulfide, could achieve a similar result awaits experimental testing. However, fougerite’s propensity to enforce redox reactions as well as its ability to interconvert redox and pH gradients is now well known (Hansen et al., 2001; Génin et al., 2005, 2006; Figure 2). Left hanging is fougerite’s possible role as a precellular inorganic non-ribosomal peptide synthetase (Bernhardt, 2019).

## 4. The fougerite nanoengine and the drive to metabolism

Our realization that prebiotic chemistry could not explain the extreme reduction of entropy involved in the emergence of life focused attention on the ferroferric DLH, green rust—now named by its discoverer, Fabienne Trolard, ‘fougerite’ (Trolard et al., 2007). Arrhenius (1984, 2003) and Arrhenius et al. (1997) were the first to see the benefits of considering green rust/fougerite (and comparable non-redox but positively charged DLHs) in this precellular context, owing to, (1) its premetamorphic abundance in Archaean banded iron formations (Arrhenius et al., 1997; Halevy et al., 2017), (2) its propensity to selectively absorb anions where, in the 2D interlayers, the effective concentrations are increased up to a million-fold (Delbrück, 1970; Arrhenius et al., 1993; Pitsch et al., 1995), (3) its “structures”; capable of dynamic agency while limiting degrees of freedom (Pitsch et al., 1995; Kolb et al., 1997), (4) its potential, sited within and as a membrane separating two strongly contrasting solutions, to respond to environmental perturbations for the sake of continued growth, *cf.*, Mitchell, 1959), and (5) such arrangements might provide the governance required for the emergence of ordered reproduction (Popov, 1999; Arrhenius, 2003; Greenwell and Coveney, 2006; Galimov, 2014; Endres, 2017; Branscomb et al., 2017; Cartwright and Russell, 2019; Gribov et al., 2021). We might add the speculation, derived from other DLHs, that its variable pattern of cations could affect the configuration of any nucleic acids produced in the system, eventually resulting in a functional polymeric sequence of nucleic acids to govern the established metabolisms (Erastova et al., 2017; Grégoire et al., 2018).

That fougerite is conformationally flexible and responds reversibly and interactively to environmental pH and redox conditions as Génin et al. (2005, 2006), Ruby et al. (2010) demonstrate also supports the hypothesis that fougerite is the precursor to the ‘free energy’/disequilibria-converting enzymes involved in conformational cycling (Nitschke et al., 2022; Equation 4):


(4) $$\begin{array}{l} {\text{Fe}}^{2+}{\;}_{4}{\text{Fe}}^{3+}{\;}_{2}{\left(\text{OH}\right)}_{12}{\text{CO}}_{3}.3_{H2}O+{2H}^{+}\left(\text{re}\right)\text{protonation}\sim \text{reduction}\Leftrightarrow \\ {\text{Fe}}^{2+}{\;}_{2}{\text{Fe}}^{3+}{\;}_{4}{\left(\text{OH}\right)}_{10}{\text{CO}}_{3}.3_{H2}O+\left(H_{2}O_{2}\right)\text{deprotonation}\sim \text{oxidation} \end{array}$$

a view warranted in consideration of the remarkable experiments of Hansen et al. who demonstrate the power of fougerite to effect the ready reduction of nitrate to ammonia—a process involving the addition of eight separate electrons to the initial nitrate as fougerite is reduced to magnetite (Hansen et al., 1996; Génin et al. 2008; Russell, 2018; Asimakidou et al., 2020). Moreover using similar conditions, Gerbois et al. (2014) demonstrate the reduction of nitrite to NO, N_2_O, and N_2_. These capabilities demonstrate an enzyme-like agency of fougerite in out-of-equilibrium geochemical systems—engineering conversions not only comparable to the nitrate and nitrite reductases, but also comparable to the enzymes such as methane monooxygenase, aminotransferase (transaminase), and acetyl phosphatase, an inorganic phosphoesterase, and, perhaps, a non-ribosomal peptide synthetase (Russell, 2018; Barge et al. 2019; Bernhardt, 2019; Branscomb and Russell, 2019; Duval et al., 2019; Huang, 2019, 2022; Wang et al., 2019).

An accompanying nickel-bearing iron sulfide mineral, mackinawite, is also planar conducting and can act as a hydrogenase (H_2_ → 2ē), ferredoxin, carbon-monoxide dehydrogenase, and acetyl coenzyme-A synthase (Hudson et al., 2020). Together, these are the conversions required to make the first ordered steps to autogenic life fed by H_2_, CO_2_, CH_4_, HNO_3_^−^, HNO_2_^−^, NO, and PO_4_^3−^ with an ambient steep proton gradient (Russell and Martin, 2004; Schoepp-Cothenet et al., 2013). Experiments grounded in the submarine alkaline vent model for life’s emergence have largely demonstrated that these conversions—this “sucking of order” from the environment (Schrödinger, 1944)—had the capacity to get autotrophic (self-ordering, self-sufficient, self-sustaining, though not self-referencing) metabolism started (Russell et al., 2003; Nitschke et al., 2022).

Having some similar properties to enzymes, these minerals or their macromolecular precursors should give us a better understanding of biological phenomena (Smith, 1986; Branscomb and Russell, 2019). Indeed, the jarring conclusion is forced that only these two minerals together can execute most of the disequilibria conversions required by the first ordered steps to autogenic life fed by H_2_, CO_2_, CH_4_, HNO_3_^−^, HNO_2_^−^, NO, and PO_4_^3−^ with an ambient steep proton gradient (Barge et al. 2019; Hudson et al., 2020). In this view, the rates of synthesis would be governed independently of variations and fluctuations in chemical concentrations and pressure through the viscosity of water ‘trapped’ in the confined spaces of the interlayers, which consequentially severely restrict the degrees of freedom of the system (Kolb et al., 1997; Astumian, 2007; Muñoz-Santiburcio and Marx, 2017; Branscomb and Russell, 2019). Furthermore, although fougerite is a 2D mineral, motions within the interlayers would be generally restricted to 1D as the 0.56-nm iron-to-iron hopping rate to next-nearest neighbors is ~ 10^10^ s^−1^ at standard temperature and pressure (STP), 3 orders of magnitude faster than those of the other two symmetry-unique hops, thus imposing vectorial flow, as in modern cells, but through the ‘green rust’ interlayers (Wander et al. 2007). This electron tunneling activity would tend to pull the more laggardly protons in single file in the hydrous interlayers by the Grötthuss mechanism (Muñoz-Santiburcio and Marx, 2017).

## 5. Chemical garden setting

Fougerite, acting as the first nanoengine driving emergent metabolism, has to be mounted and secured in the inorganic membrane such as to cater for, and feed, order-for-order exchanges while concomitantly allowing for an entropy increase *via* waste disposal. In an attempt to resolve how this might turn out to be, we return to consider the natural chemical garden membranes comprising this ferroferric-carbonate DLH and subordinate iron sulfides, further buttressed, perhaps, by silica and/or greenalite (Mielke et al., 2011; Russell et al., 2013; McMahon, 2019; Tosca et al., 2019; Barge et al., 2020, 2015a; Rasmussen et al. 2021). These are the minerals comprising the Hadean to Archaean banded iron formations (Halevy et al., 2017; Tosca et al., 2019), which are presumed to be the overspill of hydrothermal exhalations (Russell, 1975; Pons et al., 2011; Mloszewska et al., 2012).

As usually understood, crystal-hydrate gardens are self-ordered structures driven by the osmotic flow of the external alkaline water across a spontaneously precipitated semipermeable barrier (inorganic membrane) drawn inward by the high concentrations of a hydrous acidic salt as it dissolves in its water of crystallization (Leduc, 1911; Cartwright et al., 2002; Barge et al. 2012, 2015b). Growth is generally limited to tens of minutes to a few hours by the initial crystal’s mass as a result of the time taken to approach equalization of the ionic (and thereby the hydrostatic) pressures as inhibited by water stiction (Ding et al., 2016). Perret (1960) suggests that the spontaneous occurrence of expanding systems in a non-living environment such as a chemical garden might mark the first step toward the evolution of living organisms. However, in the case of the hydrothermal gardens, the expansion would mostly be the result of injection of alkaline into an ambient acidulous solution rather than osmosis (Russell et al., 1989, 1994; Russell, Hall, 1997; Mielke et al., 2011; Barge et al., 2015a). The chemical garden-like spires would continue to develop incrementally as the internal fluid perforates or breaks through, mostly at the top where the membrane is the thinnest, and reacts with the ambient fluid to produce a further segment (Barge et al., 2015b). The flow in this case is not osmotic, but ‘chemiosmotic’, where the inward transmission of individual protons via the Gröthuss mechanism (Muñoz-Santiburcio and Marx, 2017) is ~ 6 orders of magnitude faster than the outward velocity of hydroxyl ions as calculated for iron monosulfide membranes in the microfluidic experiments of Vasiliadou et al. (2019).

Extrapolation from similar microfluidic experiments involving chemical garden-like membranes comprising fougerite, as well as subsidiary mackinawite nanocrysts, is expected to reduce these external protons to hydrogen, and reduce carbonate to carbon monoxide and carboxylic acids; nitrate and nitrite to nitric oxide and ammonium; and furthermore, that the ammonium ion would aminate the carboxylic ions to the ‘short’ amino acids such as glycine, alanine, aspartate, serine, ornithine, and lysine (Hafenbradl et al., 1995; Huber and Wächtershäuser, 1998; Grégoire et al., 2016; Barge et al., 2019). Furthermore, there is some evidence to suggest that such amino acids would condense to short peptides within the confines of the interlayers of the fougerite and other DLHs where most of the water is not free, but is bound to the interior walls or even on their outer surfaces (Huber and Wächtershäuser, 1998; Rode, 1999; Huber et al., 2003; Grégoire et al., 2016, 2018; Erastova et al., 2017; Muñoz-Santiburcio and Marx, 2017; Branscomb and Russell, 2019; Rimola et al., 2022). In broad support of this view, Holden et al. (2022) show, using the electrospray mass spectrometry, that a substantial reduction in water activity does drive the condensation of glycine and alanine to dipeptide and, in droplet fusion reactions, protonated tri- and tetra-glycines. Additions of single glycines thereafter produced Gly_6_, an introduction to a peptide ‘world’ (Holden et al. 2022). Moreover, Boigenzahn and Yin (2022) demonstrate the condensation of glycylglycine to oligoglycines driven by trimetaphosphate at low water activities (*cf.* Yamagata et al., 1997).

## 6. A peptide world sequestering inorganic anions with improvement in reproduction

The formation of peptide isomers on the microsecond timescale within the interlayers on fougerite could further support the potential role of confined-volume systems in abiogenesis. That is, by membrane, cell-wall, and biofilm-like structures built from materials generated on site rather than from random organic molecules supposedly delivered haphazardly to the mound from Fischer–Tropsch reactions and the like, generated remotely at depth in the crust (Muñoz-Santiburcio and Marx, 2017; Holden et al., 2022; Römling, 2022). The addition of amino acids and short peptides to the inorganic membranes renders the chemical gardens significantly more durable (Russell et al. 1994; McGlynn et al. 2012; Barge et al., 2019; Hooks et al., 2020; Flores et al., 2021; Borrego-Sánchez et al., 2022). Moreover, as so generated, the backbone amides in short peptides would prove irresistible to inorganic ions and complexes through hydrogen bonding in the membrane—sequestering them to make enzyme-like structures such as hydrogenase and ferredoxin analogs (Nitschke and Russell 2013; Nitschke et al. 2022). Furthermore, being attractive to each other, they can produce robust peptide membranes involving the same ions—the beginning of the organic takeover (Zhang et al., 1993; Milner-White and Russell, 2005, 2008, 2011; Maury, 2009; Bianchi et al., 2012; Zhang, 2012; Kandemir et al., 2016).

Baranov et al. (2016) note that flexible linear peptides would have more structural uses and functions than cyclic molecules in the first stages of life. Moreover, Popov (1999) emphasizes that the peptide folding itself is a nonlinear non-equilibrium thermodynamic process. Intriguingly, H^+^-PPases boast of a phosphate-binding site, a protein loop (P-loop) homologous with that of ATPases, that sequesters phosphate. And just such a peptide has since been assembled in the laboratory from a mixture of simple amino acids rich in glycine, whereby its flexible backbone is shown to cosset and sequester phosphate with two of the three main chain NH groups comprising the glycine-rich peptide backbone, that is, through hydrogen bonds to the phosphate ion which thereby bridges it to take the concave form (Milner-White and Russell, 2008; Bianchi et al., 2012).

The discoveries that both bacteria and archaea have prion-like domains allow the consideration of prions being pre-LUCA (Prusiner, 1998; Zajkowski et al., 2021). It is notable that uncoded peptides are self-recognizing and tend to arrange themselves as parallel 𝛼-sheets that can spontaneously convert into the more stable and insoluble amyloid 𝛽-sheet by plane flipping (Armen et al., 2004; Milner-White et al., 2006; Hayward and Milner-White, 2008, 2021; Milner-White, 2019). Such self-propagating and temperature-resistant sheets are much stronger than lipids and have the potential to act at the emergence of life in such roles as cell membrane/cell walls and biofilm analogs (Kosolapova et al., 2020). Furthermore, comprising membranes their backbones can sequester metals and phosphate without reliance on the side-chain order (Zhang et al., 1993; Milner-White and Russell, 2005; Childers et al., 2009, 2010; Maury, 2009; Greenwald and Riek, 2010; Li et al., 2010; Goodwin et al., 2012; Milner-White, 2019). Other short peptides that do involve side chains have shown similar or superior mastery over metal-ion chelation and, thereby, agency (Aithal et al., 2023; Timm et al. 2023).

Of course, the popular view is that lipids constituted the first organic membranes but there is no theoretical or experimental evidence to suggest how they would be produced in the protometabolic system autotrophically at plausible rates and temperature. What use would they have beyond acting, as they do today, as membrane fillers and lubricants? After all, in contrast to lipids, peptides and amyloids are, (i) interactively cooperative with other ions, (ii) stronger as in their involvement in a web-like role in cell walls, strong enough even to contain turgor pressures (Kandler and König, 1998; Desvaux et al., 2006), (iii) can be seen to have roles extending to the enzyme structure, proteins, and cofactors, (iv) as well as much of the membrane, and (v) as prions they seemingly offer autonomous and ‘intentional exploration’ of space and time for similar disequilibria (Chernoff, 2004; Lupi et al., 2006; Maury, 2009; Coca et al., 2021; Jheeta et al., 2021; Zajkowski et al., 2021).

**Figure 3:**
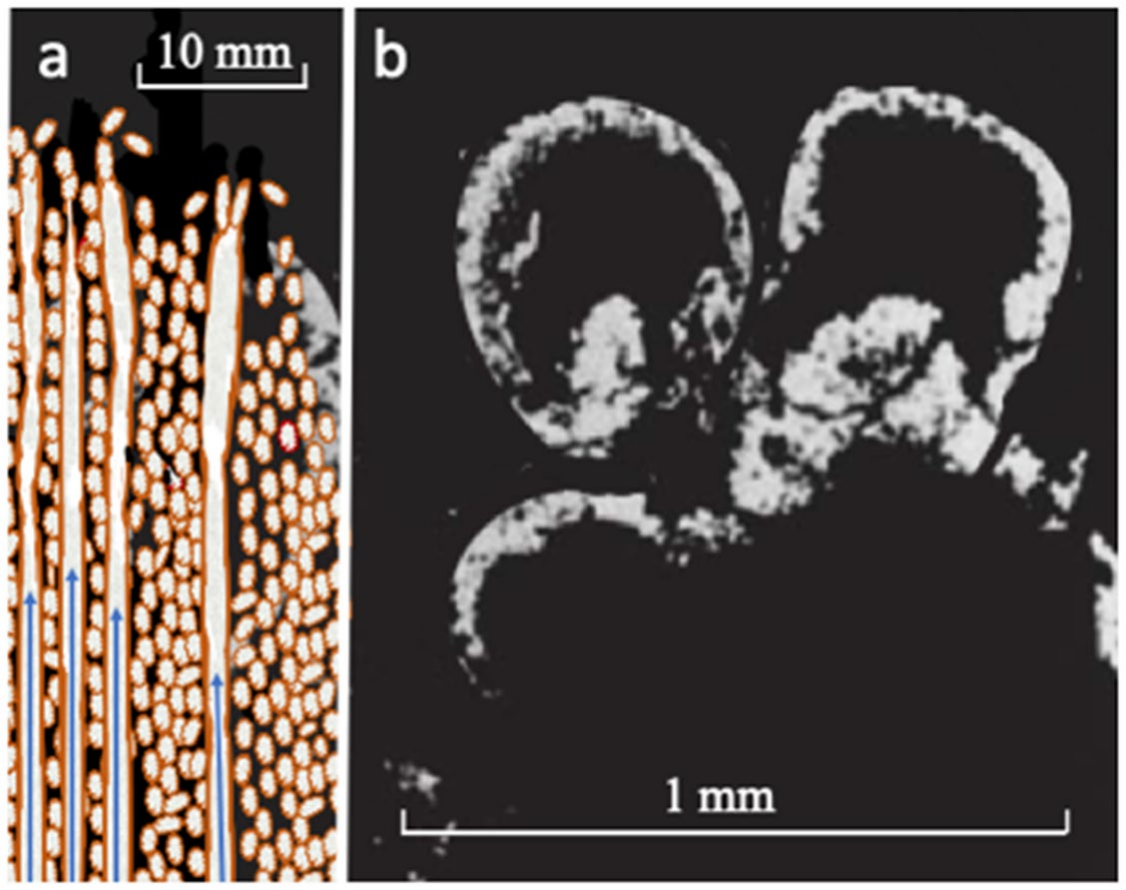
**(A)** Sketch of chemical garden spires comprising ferroferric oxyhydroxide and minor iron sulfide that spontaneously precipitate and continue to grow through injection at alkaline/acidic interfaces (Barge et al., 2015a, b; Helmbrecht et al., 2022; Akbari and Palsson, 2022b; see Figure 1). Short peptides produced in the fougerite interlayers are presumed to be gradient-driven vectorially through the fougerite nanocrysts from outside to the spire’s inner surface where they are hypothesized to form metal-dosed organic films signified here by brown color (*cf.*, Oda and Fukuyoshi, 2015; Römling, 2022). Within certain limits of externally applied disequilibria, the fougerite or similar DLHs act like an enzyme, in that organic production rates remain constant in spite of fluctuations in supply (Pitsch et al., 1995; Hansen et al., 1996; Kolb et al., 1997; Branscomb et al., 2017). Continuation of the process leads to spalling and entrainment and eventual crowding at the growing spire’s tip where necking-off produces organic/inorganic cells that gravitate to depth in a geode (also brown; Russell and Martin, 2004): **(B)** natural chemical garden sulfide bubbles by comparison in the 340 Ma Tynagh orebody (Russell and Hall, 1997).

Under alkaline vent conditions, amyloid peptides and amyloid fibrils would be expected to exude from the interlayers to produce organic molecular webs adhering to the spire’s inner walls as metal-dosed organic films (Takahashi and Mihara, 2004; Larsen et al. 2007; Römling, 2022; Figure 3). Subject to entrainment in the hydrothermal updrafts, some of this amalgam is likely to spall off in the general direction of flow, eventually crowding and necking to form offspring capable of interacting and sharing the peptide film (Milner-White and Russell, 2005; Larsen et al. 2007; Greenwald and Riek, 2010; Greenwald et al., 2018; Römling, 2022). Yet, it is admitted that this speculation falls well short of how prions in membrane or cell-wall microbes could segue to a peptide–nucleotide world governing metabolic pathways and the reverse Krebs cycle (Gallardo and Rodhe, 1997; Schumann and Huntrieser, 2007; Carey et al. 2016; Weiss et al. 2016; Bromberg et al., 2022; Harrison et al., 2022; Lane, 2022; Palmeira et al., 2022). For this, we are forced to consider the emergence of an albeit imperfect genetic governance to get life into its historical and present role.

Furthermore, Abel and Trevors (2006) provide a cogent argument against the assumption that the complexification of peptide- and prion-assisted metabolisms is enough to initiate vertical evolution without the insertion of a rule-based physicochemical program. In other words, we have to face up to the introduction of a material program, which makes life distinct in being able to defend itself by exerting an autonomous choice, if surprised by external alterations beyond itself (Kordium, 2021). This is in contrast to ALL other simple self-ordering phenomena driven by the many varieties of known disequilibria (Russell et al., 2013; Ramstead et al., 2019).

However, there is at present no clearcut path from a putative fougerite–mackinawite–peptide reproducing system to a nucleotide-based replicative one.

## 7. Genome programming—from ordering to organization

Noting the main dilemma in the origin-of-life research, Freeman Dyson famously suggests that life must have originated twice “with two separate kinds of creatures, one kind capable of metabolism without exact replication and the other kind capable of replication without metabolism” (Dyson, 1986). Metabolism’s creature seems to emerge through the synthesis and reproduction of amino acids and even peptides but has nowhere to go, while the replication creature is supposedly born whole in an age of information, yet cannot find the wherewithal to ‘be’. These two creatures each have their champions and the regrettable outcome is the erection of an intellectual wall built between the ‘computing replicationists/geneticists’ and the ‘engineers of metabolism’. What to do?

The ‘metabolists’ do need to find geologically informed ways to synthesize the nucleotides indispensable to making a code. The ball is in our court. Just how information was introduced early into the engines of metabolism is the ‘hard problem of life’ (Walker and Davies, 2017; Wong and Prabhu, 2023). From a crystallographic perspective, we might start with the size and shape, recalling Erwin Schrödinger’s classification of the gene as an aperiodic crystal (Schrödinger 1944). Moreover 2 years later, Linus Pauling (1946) promulgated his views on the importance of complementarity of molecular shape as determinants of their interactions (Pauling, 1946), originally considered as the “lock and key” requirement for molecular interactions (Fischer, 1894) and the “side chain theory” of Ehrlich (1901). These articles set the scene for the self-assembly hypotheses of: (1) Dounce’s (1953) nucleic acid template hypothesis, whereby the ribonucleic acids synthesized on the gene templates would, in turn, become templates for protein synthesis, (2) Gamow’s (1954 and Gamow and Yčas, 1955) double coding hypothesis, in which “amino acid residues in proteins are selected by independent triplets of nucleotides,” and (3) Nirenberg and Leder’s (1964) “affinity method.”

Woese et al. (1966) picked up on these ideas, framing the issue in terms of “whether or not amino acid-oligonucleotide steric interactions play or have played a role in determining these assignments, and if so, to what extent?” Their resounding and “essentially unavoidable conclusion” is that “codon assignments manifest an underlying codon-amino acid pairing”; a conclusion still resonating today (Woese et al., 1966; Russell et al., 2003; Yarus et al., 2005, 2009; Yarus, 2021; Harrison et al. 2022). Moreover, it leads to Massimo Di Giulio’s (2008) hypothesis of an extension of the coevolution theory for the origin of the genetic code.

While these theories have been widely entertained, experimental exploration is limited. One notable success is due to Mellersh and Wilkinson (2000) who demonstrated, for example, how “polyadenylic acid immobilized on silica gel stereoselectively binds L-lysine from dilute aqueous solution”… and so facilitates “subsequent amide bond formation” (Russell et al., 2003). At the same time, Levy and Ellington (2003) expand upon their ideas regarding peptide-templated nucleic acid ligations. Mike Yarus (2017) confirms these affinities by demonstrating that the RNA–amino-acid interface logically relates triplets to the side chains of particular amino acids, concluding that “peptides may have been produced directly on an instructive amino acid binding RNA” (Yarus, 2017). We should also note the possibility that, given the degeneracy in the genetic code, the progenitors of the earliest genetic code were codons with four bases (or more)—the tessera codes of Baranov et al. (2009) and Gonzalez et al. (2012, 2019). Yarus (2011) also sees a way of “getting past the RNA world”—a world that never was according to Yockey (1995), Kurland (2010), and Wills and Carter (2018).

Wächtershäuser (1990), Martin and Russell (2007), and Harrison and Lane (2018) have made attempts at a “progression” but it might be argued, to use Stanley Miller’s apothegm, that these erections are nothing more than “paper chemistry” (Hagmann, 2002, but see Polanyi (1962, p. 165) for a thoughtful defense of such ‘speculations’). Abel and Trevors (2006) attempt to discipline the “metabolists,” by pointing out that, absent a program, metabolic cycling is doomed to docile repetition as long as their particular driving disequilibria last, as per the laws of chemistry and physics. To animate metabolism and make it reflexive, we cannot expect complexification *per se* to answer the conundrum (Abel, 2011). The workings of life have to be understood in terms of their entirety and as Abel (2011) emphasizes, work itself “entails more than spontaneous phase transitions.” Once metabolism’s disequilibria-converting engines are up and running, to allow the system as a whole to progress and evolve they all must be algorithmically directed, and continually replaced (Trixler, 2021). Moreover, to last, any product stemming from the alkaline mound has to have its use as a component part of each metabolic engine; has to pay its way or be discarded (Branscomb et al., 2017). There seems nothing for it but to seek a non-ribosomal peptide synthetase that includes nucleotides in its structure (Kleinkauf and von Döhren, 1996; von Döhren et al., 1999; Fischbach and Walsh, 2006).

Attempts to assail this conceptual wall have been less than successful. However, just this year some ‘cracks’ have appeared on the metabolist-*cum*-chemical side. The Nick Lane–Stuart Harrison group at the Department of Genetics, Evolution and Environment, University College London, look to how randomly synthesized nuclear monomers could become involved in the very basis of metabolism—namely, as nucleotide catalysts in CO_2_ hydrogenation and in amination of carboxylates to amino acids (Harrison et al. 2022; Palmeira et al., 2022; Pinna et al. 2022). This forward-looking approach can explain why ATP is universally conserved across life (Pinna et al., 2022). Moreover on this side, in a series of experiments, Joseph Moran’s group at the Institut de Science et d’Ingénierie Supramoléculaires, Strasbourg, France, demonstrates the likely networks followed by the earliest non-enzymatic metabolic pathways, for example, metal-ion transaminations (Mayer et al., 2021), the centrality of iron in catabolic as well as anabolic processes (Muchowska et al., 2019, 2020), and the abiotic conversion of aspartate to orotate and further reactions to produce all three of the pyrimidine nucleobases in water at 60°C catalyzed by a variety of metal ions along with an oxidant (Yi et al., 2022). Furthermore, Müller et al. (2022) explore ‘palaeochemistry’ in their search for a plausible scenario of an RNA–peptide world, while Akbari and Palsson (2022a) formulate how metabolic homeostasis and cellular growth might arise in the acetyl coenzyme-A pathway and the reductive TCA cycle, and Helmbrecht et al. (2022) demonstrate the accumulation of RNA in amakinite/fougerite chemical gardens.

How much of this might progress in the precellular DLH world? Pitsch et al. (1995) demonstrate how a weakly alkaline 20 μM solution of glycolaldehyde phosphate can, once absorbed within M^2+^/M^3+^ oxyhydroxide interlayers of green rust, be transformed to hexose- and pentose-phosphates—the latter structurally related to the sugar phosphate units in RNA. Moreover, Krishnamurthy et al. (1996) demonstrate that the ‘alternative’ nucleic acid pyranosyl ribose-2,4-phosphate, the near-planar sugar phosphate structure formed in similar conditions, is a nucleic acid with exceptional base-pairing properties (Eschenmoser, 1994). Krishnamurthy et al. (1996) show that, on introducing formaldehyde and glyceraldehyde phosphate into the DLH interlayer, 40% of the product consists of pyranosyl ribose.

**Figure 4:**
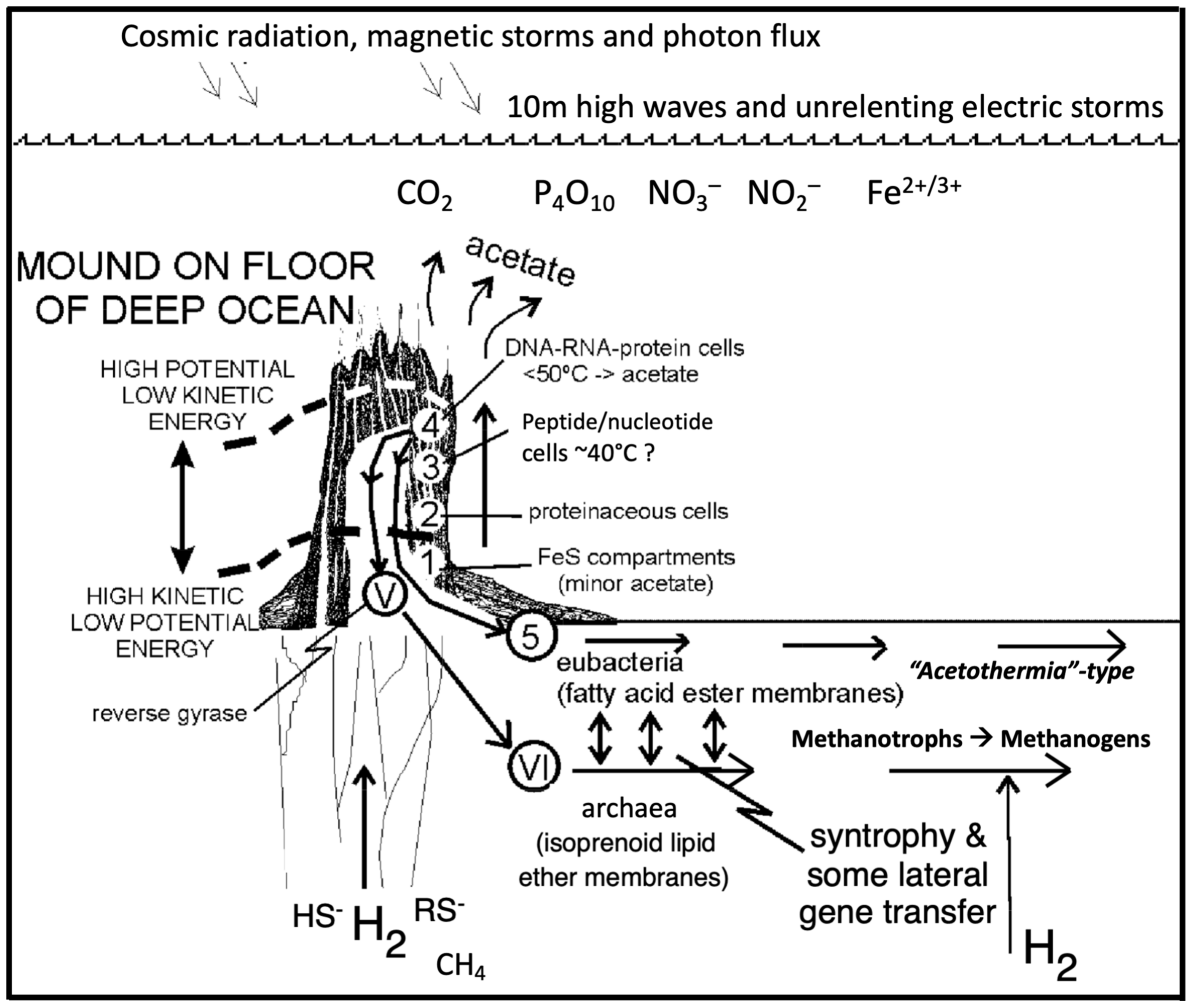
Emergence of the deep biosphere. Autotrophic life emerges and rapidly infects a hydrothermal alkaline mound (Figure 1) and differentiates interdependently into the precursors of the bacteria and archaea, grows by expansion downward and laterally into the surrounding sediments and serpentinizing ocean crust, thus initiating the deep biosphere, a hypothesis now broadly supported by recent research (Fones et al., 2019, 2021; Boyd et al., 2020; Berkemer and McGlynn, 2020; Leong et al., 2021; Colman et al., 2022). Numbers 1–3 relate to life’s emergence, while 4 marks the supposed point of differentiation of the archaea and the bacteria. Roman numerals V–VI mark evolutionary stages of the archaea, and number 5 shows the stages of evolution of the bacteria in the deep biosphere (redrawn from Russell and Arndt, 2005).

Perhaps, the secret of “The First Cell,” the title of Azra Raza’s book, which refers to the first cancer cell in an oncology case, is also the secret of the very first cell (Raza, 2019; Marshall, 2021; see Szent-Györgyi, 1968). According to Bernhardt and Patrick (2014), genetic code evolution started with the incorporation of glycine, followed by other small hydrophilic amino acids. Certainly, once the genetic code is sophisticated to the extent of being able to take on surprises and make choices and generate novel information (Marshall, 2021), there can be a rush to infest the entire hydrothermal mound. And life is ready for its diaspora. While relatively slow to colonize the ocean crust at first, as an entropy generator able to pick up any stray leftovers from other disequilibria generating systems, life takes over the surfaces on, and within, our planet eventuating in photosynthesis (Russell and Arndt, 2005; Figure 4).

## 8. The taproot and first branch of the evolutionary tree

In the present AVT, life is rooted in methanotrophic acetogenic microbes respiring nitrate (Nitschke and Russell, 2013). The taproot itself is grounded in fougerite, which sees to the harnessing of ambient H_2_, CH_4_, and CO_2_ driven by the natural proton motive force and respiration of oxidized nitrogen entities (Barnes and O’Neil, 1969; Neal and Stanger, 1983; Ducluzeau et al., 2009, 2014). Once this system is up and running at the outer margins of the submarine alkaline hydrothermal mound, its requirements are, nevertheless, highly constricting. The evolutionary breakout (‘break in’) comes with metabolism’s discovery of how to survive on the much reduced free energy from readily available H_2_ and CO_2_ within the mound itself. We speculate that Christian Schöne et al.’s (2022) exciting finding of the facile conversion of an archaeal methanogen to a carbon-monoxide-dependent acetogen through the removal of cellular function could be read as an indication of how “reverse methanotrophy” segued to an acetogenesis, perhaps related to the differentiation of the progenote into the archaea and bacteria (Russell and Nitschke, 2017; *cf.*, Lyu et al., 2022). This admittedly contentious suggestion sees a parallel in human society having to burn hydrocarbons while ‘waiting for nuclear fusion(s)’.

## 9. Escape from the mound and the founding of the deep biosphere in serpentinizing ocean crust

Entrainment of the earliest microbes in a hydrothermal effluent to the ocean would lead to their immediate starvation. Thus, it is surmised that the only survivors are those who are caught up in an involuntary and random growth and expansion in a downward front to inaugurate the deep biosphere (Pedersen, 1993; Parkes et al., 1994; Parkes and Wellsbury, 2004; Russell and Arndt, 2005; Glasby, 2006; Schrenk et al., 2013). In such conditions, the ‘law of natural rejection’ would see all but the most efficient cells or cellular cooperatives die-off.

In the oceanic crust itself, these survivors would have missed the profusion of the mound and been drastically thinned out and stripped of non-essential genes (Fones et al., 2019, 2021). Fones et al. (2021) make the cogent argument that the absence of CO_2_ in this new environment drives the adaptation of methanogens to generate their own. Discrete ocean downdrafts are another source of CO_2_. Yet, the remaining feedstocks, while restricted, are otherwise not so different in the serpentinizing throat and ultramafic surrounds, perhaps also supplying the electron-donating H_2_, CH_4_, HCOO^−^, CO, and CH_3_COO^−^ (Windman et al., 2007; White et al., 2020). The latter four entities also supply the substrate carbon (White et al. 2020). Such alkaline fluids are known to support microbial nitrate and nitrite bacterial reduction (Albina et al., 2021).

Colman et al. (2022) offer a window into the effect of such conditions in their exhaustive study of the Semail ophiolite. Not only are reproduction rates much diminished but genetic diversity too is “streamlined” for survival (Colman et al., 2022). Furthermore, syntrophy, gene swapping both within and across domains, and the sharing of nutrients keep the microbiome operating (Wolin, 1982; Russell and Arndt, 2005; Tiago and Veríssimo, 2013; Kohl et al., 2016; Brazelton et al. 2017; Suzuki et al., 2017, 2018; Colman et al. 2022).

Extrapolating across the 4 billion years since life’s onset, we gauge from Colman et al. (2022) that the autotrophic acetogenic analogs comparable to what they term type II *Acetothermia* would survive displacement from the mound into the hyperalkaline waters in equilibrium with incipient serpentinization. These types of *Acetothermia* employ an archaeal-like carbon-monoxide dehydrogenase and ferredoxin-based complexes to achieve acetogenesis. Moreover, they have the capacity for respiratory growth using nitrate (Youssef et al., 2019). Other bacteria revealed by metagenomics are the sulfate-reducing bacteria (Brazelton et al., 2017; Rempfert et al., 2017; Templeton et al., 2021).

Although apparently missing from the Semail ophiolite, sequences of an anaerobic Methanotroph group 1 (ANME-1) have been identified in the serpentinization-driven alkaline Cabeço de Vide aquifer in Portugal, and unclassified anaerobic methanotrophic euryarchaeota (ANME) MAG are recorded from Lost City (Tiago and Veríssimo, 2013; Nothaft et al., 2021). However, suggestions that these findings provide an inkling of support for the methanotrophy-first speculation of Nitschke and Russell (2013) are put on hold by the likelihood of the present-day “contamination from surface waters” (Merkel et al., 2013; Postec et al., 2015; Trutschel et al., 2022).

## 10. The submarine alkaline vent theory put to the test

Experiments on, and analyses of, AVT look to an evolutionary tree with its deepest roots in cosmogenesis—yet reaches upward and along the lowest branches of the acetyl coenzyme-A and an incomplete reverse TCA cycle (Nitschke and Russell, 2010; Russell et al., 2013; Carroll, 2016).

Findings and some predictions of AVT are that:

1. A natural proton disequilibria measuring between 2 to 5 pH units imposed across an FeS membrane separating a hydrogen-bearing alkaline solution from a carbonic ocean drives the hydrogenation of the CO_2_ to formate/CO (Russell and Hall, 1997; Branscomb and Russell, 2013; Vasiliadou et al., 2019) is verified (Hudson et al., 2020), although most of the iron in the membrane is now argued to be in fougerite/green rust rather than in FeS (Russell et al., 2013).
2. An expectation of AVT is that methane derived from the lower mantle and entrained in the alkaline hydrothermal fluid will oxidize to a methyl group within green rust (Nitschke and Russell, 2013), itself (re)oxidized by NO and nitrate provided through …
3. appreciable volcanic and hurricane cloud-to-cloud lightning, bolide impacts, and photochemistry from the CO_2_ + N_2_ atmosphere dissolved in the ocean as CO_2_, NO_3_^−^, and NO_2_^−^ (Mancinelli and McKay, 1988; Gallardo and Rodhe, 1997; Kasting and Siefert, 2001; Ducluzeau et al., 2009; Wong et al., 2017; Navarro-González et al., 2019).
4. Although conclusion 3 is challenged by Ranjan et al. (2019), the view that the nitrogen oxides were present in the Earth’s early atmosphere and that their derivatives also invaded the ocean is now strongly reinforced by Buessecker et al. (2022) who suggest green rust to be responsible for carrying NO to depth bound as nitrosyl (and see Heays et al., 2022; Nishizawa, 2022) …
5. in turn supporting the denitrifying methanotrophic acetogenesis hypothesis as the first pathway to life, predating the more demanding acetyl coenzyme-A pathway (Nitschke and Russell, 2013; Russell and Nitschke, 2017) …
6. and that the amination of pyruvate and oxalate to alanine and glycine which can be accomplished *via* fougerite (Nitschke and Russell 2013) is verified by Barge et al. (2019, 2020) …
7. while the condensation of amino acids to peptides within the 2D interlayers of peptides has support from Muñoz-Santiburcio and Marx (2017), Erastova et al. (2017), Grégoire et al. (2016, 2018), and Holden et al. (2022).
8. Fougerite interlayers are capable of dampening externally imposed disequilibria to produce an enzyme-like flux-force linearity to interactants (Branscomb and Russell, 2019), …
9. although indications of binding, binding-change, and disequilibria-conversion mechanisms (Russell, 2018) await experimentation using the operando X-ray absorption spectroscopy and allied techniques (Fracchia et al., 2018).
10. That the deep biosphere—initially the serpentinizing Hadean ocean crust—is first populated with respiring denitrifying methanotrophic archaea and acetogenic bacteria from their point of ‘origin’ (the progenote) in a submarine alkaline hydrothermal mound (Russell and Arndt, 2005; Nitschke and Russell, 2013; Ménez, 2020) is supported by circumstantial evidence; e.g., the bacterium *Acetothermia* is capable of respiration with nitrate (Fones et al., 2019, 2021; Youssef et al., 2019; Boyd et al., 2020; Colman et al., 2022) and an archaeon methanotroph, *cf.* ANME-1 (Tiago and Veríssimo, 2013; Brazelton et al., 2017). We might imagine these progenotes happening upon new locations with high concentrations of dissolved ions and gases, carried there passively by percolating solutions.

## 11. Conclusion

The production of alkaline hydrothermal waters through the serpentinization of mafic to ultramafic rocks, as introduced by Ivan Barnes et al., underpins AVT (Barnes and O’Neil, 1969; Barnes et al., 1972; Russell et al., 1989; Macleod et al., 1994). These authors also figure in the description of the Lost City vents discovered in 2000 (Kelley et al., 2001). The serpentinizing system that produces such alkaline submarine emissions has, through a reading of Cottrell (1979), since been described in terms of a disequilibria- (‘free-energy’-) converting cracking engine (Russell et al., 2013). While AVT originally assumed a sulfide mound to be generated at the spring-to-ocean interface, it became apparent that the double-layer oxyhydroxide green rust (fougerite) would be the major ferroferric precipitate along with some sulfide and Mg-rich clays and silica (Russell and Hall, 1997; Russell and Arndt, 2005). Because of its physicochemical flexibility, this macromolecular 2D mineral could also be considered the necessary nanoengine/protomotor enzyme—in this case to convert the disequilibria between H_2_ + OH^−^ + CH_4_ and CO_2_ + NO_3_^−^ + H^+^ to the rudiments of the denitrifying methanotrophic acetyl coenzyme-A pathway—the interlayers acting as precursor metabolic channels toward further downstream organic synthesis (Nitschke and Russell, 2013; *cf.* Srere, 1987).

The current AVT for life’s emergence has it that fougerite-rich hydrothermal electrochemical gardens (fine chimney stalks and spires) mark the precellular stage [Russell et al., 2005, 2013 (Figure 3B); Barge et al., 2015b; Chin et al., 2020; Nitschke et al., 2022 (Figure 3)]. We imagine a well-ordered convective alkaline updraft feeding reductants to the also well-ordered macromolecular fougerite comprising the growing spires and chimneys, which in turn allows a well-ordered infiltration and vectorial flow of protons and anions from the ocean directed through the interlayers (Borrego-Sánchez et al., 2022). The result is an effusing tangle of organic molecules probably dominated by peptides to coat the inner wall of the spires (Akbari and Palsson, 2022b; Römling, 2022). Portions of this organic film spall off and are entrained in the flow, along with other organic molecules released by delamination and/or by diagenetic alteration to magnetite at depth in the mound. Some of this organic material may be ‘attracted’ to form cellular structures that are capable of sensing and responding to oscillations and fluctuations in supply and perhaps prove eventually to be self-sustaining, and, on the development of emerging genetic algorithms, would be able to make choices and generate novel information and rapidly infect the mound (Cain, 1949; Marshall, 2021).

Thus, beginning with the insights of Ivan Barnes et al., we argue that:

1. Serpentinization is the inescapable response of the Hadean Earth’s ultramafic crust to the circulation of ocean water. Moreover, it is equally inescapable that the preorganized macromolecule fougerite is a prerequisite for the dissipation of the disequilibria resulting from the return of serpentinite’s effluent to its original source, the Hadean carbonic ocean (Tatzel et al., 2022).
2. Just as cracks in the Hadean ocean floor are a prerequisite for priming serpentinization, so do aqueous interlayers within redox/pH-sensitive fougerite nanocrysts acquiesce to the forceful vectorial invasions from either flank of the precipitate membrane of pent-up ions, charged and uncharged fuels and oxidants, eventuating in protometabolisms (Nitschke and Russell, 2013).
3. However while fougerite may be considered to provide simple messages to govern a product, it is presumed that prion-like offspring may offer seemingly autonomous and ‘intentional exploration’ of space and time for similar disequilibria (Maury, 2009).
4. However, a second chapter on life’s emergence awaits an understanding of the paths from the earliest coherent pregenetic governing algorithms of life to the emergence of the “modern synthesis”—both barely written introductions to the subsequent well-versed chapters of life’s unitingly diversifying Gaian commonwealth where “everything is everywhere (though the environment selects)” (Baas Becking, 1931, 1934; Quispel, 1998; Abel and Trevors, 2006; Igamberdiev, 2021); *pace* (Martiny et al. 2006).

An understanding of how life both emerges and thrives requires the use of stochastic or trajectory thermodynamics—equilibrium thermodynamics is absolutely inappropriate for the task (Astumian, 2018, 2020; Bartlett and Beckett, 2019; Branscomb Russell, 2019; Horowitz and Gingrich, 2020; Feng et al., 2021; Ueltzhöffer et al., 2021). Furthermore, of course, experiments are required to test the hypothesis that self-sustaining hydrothermal ‘electro-chemical gardens’ comprising the minerals fougerite and mackinawite as nanoengines and nanoengine mountings are up to the task of engendering the earliest steps of life (Figures 2, 3). Thereafter, computer modeling and artificial intelligence beckon as ways of further resolving sequences of life’s emergence (Ugliengo, 2019; Hassabis and Revell, 2021).

## Data availability statement

The original contributions presented in the study are included in the article/supplementary material, further inquiries can be directed to the corresponding author.

## Author contributions

The author confirms being the sole contributor of this work and has approved it for publication.

## Conflict of interest

The author declares that the research was conducted in the absence of any commercial or financial relationships that could be construed as a potential conflict of interest.

## Publisher’s note

All claims expressed in this article are solely those of the authors and do not necessarily represent those of their affiliated organizations, or those of the publisher, the editors and the reviewers. Any product that may be evaluated in this article, or claim that may be made by its manufacturer, is not guaranteed or endorsed by the publisher.
